# Differences in Regional Anesthesia Utilization by Hospital Region in the United States

**DOI:** 10.7759/cureus.46795

**Published:** 2023-10-10

**Authors:** Alexander Beletsky, Morgan Currie, Jonathan Shen, Hunter Brooks, Mahesh Desilva, Nutan Winston, Rodney A Gabriel

**Affiliations:** 1 Anesthesiology, Riverside Community Hospital, Riverside, USA; 2 Anesthesiology, University of California San Diego, San Diego, USA

**Keywords:** peripheral nerve block, neuraxial anesthesia, spinal anesthesia, tsa, tka, aclr, trends analysis, regional anesthesia

## Abstract

Background: Regional anesthesia has been associated with improved postoperative outcomes. Disparities in regional anesthesia utilization exist; however, no studies have examined utilization rates as a function of hospital region.

Methods: A national hospital database (Hospital Corporation of America {HCA}) was queried for patients aged 18 years or older that received selected surgical procedure codes between January 2016 and June 2021. Surgical procedures included were total knee arthroplasty (TKA), total shoulder arthroplasty (TSA), anterior cruciate ligament reconstruction (ACLR), carpal tunnel release, total abdominal hysterectomy (TAH), open reduction and internal fixation (ORIF) of the ankle, and arteriovenous (AV) fistula creation. Regional anesthesia was defined as any form of neuraxial and/or peripheral nerve blocks. Basic summary statistics were utilized to calculate the rates of regional anesthesia (RA), and chi-squared analyses were calculated to determine significant differences in the rate of RA utilization.

Results: There were 52,068 patients included in this study, of which 2,114 (4.1%) received RA. The greatest RA rates were for TSA (5.8%), TKA (4.5), and anterior cruciate ligament reconstruction (ACLR) (3.6%), whereas the lowest RA rate was for TAH (1.1%). For the TKA cohort, the Midwest had a significantly greater utilization rate than the South or West (10.9% vs. 4.8% or 3.1%, p<0.001). The Midwest also had the highest utilization rate in the ACLR cohort (8.1%, p<0.001), TAH cohort (16.7%, p<0.001), and AV fistula cohort (6.4%, p<0.001). For the carpal tunnel cohort, the West had the highest utilization rate (11.8% vs. 8.1%, 1.1%, 0%, p<0.001). The West region also had the highest utilization rate for the ankle ORIF (7.8%, p<0.001). No significant differences were found by region for TSA (p=0.31).

Conclusion: Significant variations in RA utilization rates were found by region, with the West having the highest utilization for ankle ORIF and carpal tunnel, and the Midwest having the highest rate for TKA, ACLR, TAH, and AV fistula.

## Introduction

In the era of improved methods of data collection allowing for the construction of highly powered cohorts, the analysis of postoperative outcomes with the use of regional anesthesia (RA) as the primary anesthetic has emerged as a topic of increasing interest [1]. RA has demonstrated benefits in acute pain and persistent postoperative pain after thoracotomy or breast surgery [2-5]. Other benefits of RA include decreased intraoperative [6] and postoperative opioid requirements [7] after various surgeries [8], shorter mean times to discharge [9], improved same-day discharge rates after clavicular surgery [10], and improved patient satisfaction rates, particularly in upper extremity surgery [11].

More studies are needed that describe utilization rates of regional anesthesia. As providers [12] and patients [13] comfort with RA changes across the country, as well as the scope of regional anesthesia coverage broadening over the past 40 years [14], it is important to understand factors that may contribute to disparities in its use (i.e., hospital resources, provider training, patient comfort, surgeon support, and setting-specific variation) [15]. While a few studies have examined utilization rates of RA over time, more studies are needed to help define regional anesthesia trends across numerous surgical procedures and hospital regions (i.e., West, South, Northeast, Midwest) [16,17].

The purpose of the current study was to utilize a large national database (Healthcare Corporation of America {HCA}) to examine RA utilization rates in a procedure and hospital region-specific manner. We had two following hypotheses: (1) significant variation exists across surgical procedures and (2) statistically significant variability in RA utilization by hospital region will exist for each procedure in question.

This article was previously published as a preprint on the Research Square server on September 16, 2022 (https://doi.org/10.21203/rs.3.rs-1980696/v1).

## Materials and methods

Data registry

Prior to initiation of the study, the study was reviewed by the Riverside Community Hospitals Institutional Review Board and deemed to have certified exempt status from patient content. Institutional permission was granted to utilize a national database collecting anesthetic case data across all Hospital Corporation of America (HCA) hospitals. This is a multi-centered clinical repository collecting de-identified data inclusive of demographic, preoperative, perioperative, and postoperative variables. All data utilized in this study were de-identified prior to analysis. Variables of interest included all available demographic variables, hospital zip code (i.e., hospital region), and perioperative and postoperative variables. 

Study population and covariates

The study population was adult individuals of age 18 years or older without an upper age limit. Other inclusion criteria included receipt of a surgical procedure of interest between January 2016 and June 2021, and receipt of anesthesia (i.e., general, regional, monitored anesthesia care {MAC}, etc.) (Table 1). There were no specific exclusion criteria for this study (Table 1).

**Table 1:** Surgical procedures and procedures of interest.

Common procedural terminology	Procedure of interest
23472	Total shoulder arthroplasty
27447	Total knee arthroplasty
64721	Carpal tunnel surgery
29888	Anterior cruciate ligament reconstruction
27766, 27792, 27814, 27823	Ankle open reduction and internal fixation
58150	Total abdominal hysterectomy
36830	Arteriovenous fistula creation

The primary outcome of interest in this study was the rate of RA receipt, coded as a binary variable. The definition of RA in this study was neuraxial anesthesia (NA) (i.e., spinal, epidural) and/or peripheral nerve blocks (PNBs), inclusive of upper extremity blocks (i.e., axillary, interscalene, supraclavicular, infraclavicular) and lower extremity blocks (i.e., infiltration between the popliteal artery and capsule of the knee (IPACK), adductor canal, genicular, saphenous, popliteal). Preoperative variables included in this study were demographic factors (i.e., age, sex, race, body mass index, insurance status). Hospital regions had four distinct regions - West, Northeast, South, and Midwest - defined on a per-state basis mirroring definition provided by hospital regions [18]. The surgical procedures included in this analysis were total shoulder arthroplasty (TSA) (concurrent procedural terminology {CPT}: 23472), total knee arthroplasty (TKA) (CPT: 27447), carpal tunnel release (CPT: 64721), anterior cruciate ligament reconstruction (ACLR) (CPT: 29888), ankle open reduction and internal fixation (ORIF) (CPT: 27766, 27792, 27814, 27822, 27823), total abdominal hysterectomy (CPT: 58150), and arteriovenous (AV) fistula creation (CPT: 36830). Charlson Comorbidity Index (CCI) was used as a variable in this study, which is a model predicting 10-year survival in patients with multiple comorbidities. It utilizes age, along with various comorbidities including myocardial infarction, congestive heart failure, stroke or transient ischemic attack, obstructive lung disease, diabetes, and liver disease, among other comorbidity data. American Society of Anesthesiologists (ASA) physical classification system was also used, which is an ordinal number system from 1 to 6, with each level corresponding to patients with greater severity of disease presenting as an increasing threat to life.

Statistical analysis

A power analysis was performed modeling chi-squared analysis, assuming a “worst-case” scenario with an effect size of 0.1, equal to 0.05, power of 0.95, and three degrees of freedom, suggesting a minimal sample size of n=1717. Analysis for this project involved summary statistics of the rate of RA utilization, defined as total cases utilizing RA divided by total cases for a given CPT. On subgroup analysis, when we split cases up by region, univariate statistics involving chi-squared analyses were performed to determine if statistically significant differences existed by region for a given CPT code. Specific data definitions used in this study included “percentage with RA,” which corresponds to the percentage in a given region of the total RA delivered for a given CPT, and “percentage within region” defined as the RA utilization rate within a given region. “Percentage of total RA delivered” corresponded to what share of the total RA count was delivered by a given region.

## Results

A total of 52,068 surgical cases met the inclusion criteria in the study, with 2,114 (4.23%) of those cases receiving RA. Among the surgical techniques, the large majority of surgeries were TKA (n=29,440, 56.5%), followed by carpal tunnel release (n=8,045, 15.5%), ankle open reduction and internal fixation (n=6,319, 12.1%), and anterior cruciate ligament reconstruction (n=2,729, 5.2%). Statistically significant differences were found between regional and non-regional cohorts for sex (p=0.02), race (p<0.001), hospital region (p<0.001), Medicaid insurance (p<0.001), CCI (p=0.02), and ASA (p<0.001) (Table 2).

**Table 2:** Demographics of the study population. CCI: Charleson Comorbidity Index; ASA: American Society of Anesthesiology Physical Classification; RA: regional anesthesia; TKA: total knee arthroplasty; ACLR: anterior cruciate ligament reconstruction

Age in years (STD)		Total N/mean	RA=0 (n=49,954)	RA=1 (n=2,114)	p-Value
		61.7±14.8	61.7±14.8	61.5±14.5	0.71
Female sex, n (%)		30,597 (58.76%)	29,406 (58.87%)	1,191 (56.34%)	0.02
Black race, n (%)		5,734 (11%)	5,566 (11.14%)	168 (7.95%)	<0.001
Obese BMI, n (%)		13,938 (26.77%)	13,359 (26.74%)	579 (27.39%)	0.85
Surgical category	Carpal Tunnel	8,037 (15.44%)	7,779 (15.57%)	258 (12.2%)	<0.001
	TKA	29,438 (56.54%)	28,116 (56.28%)	1,322 (62.64%)	
	ACLR	2,728 (5.24%)	2,630 (5.26%)	98 (4.64%)	
Region	South	38,614 (74.16%)	37,220 (74.51%)	1,394 (65.94%)	<0.001
	Midwest	2,238 (4.30%)	2,064 (4.13%)	174 (8.23%)	
	West	10,438 (20.1%)	9,893 (19.8%)	545 (5.78%)	
Medicaid, n (%)		3,268 (6.28%)	31.85 (6.38%)	83 (3.93%)	<0.001
CCI		2.60	2.60	2.50	0.02
ASA		2.5±0.7	2.51±0.7	2.4±0.7	<0.001

When examining total RA anesthetics delivered by hospital region, the South region (65.9% of total) had the most RA delivered compared to the West (25.8%) and Midwest (8.2%) (p<0.01). The Midwest region had the highest overall RA utilization rate (7.8%) when compared to the West (5.2%), South (3.6%), and Northeast (0.1%) regions (p<0.01) (Table 3).

**Table 3:** RA utilization rates by procedure. TSA: total shoulder arthroplasty; TKA: total knee arthroplasty; ACLR: anterior cruciate ligament reconstruction; ORIF: open reduction and internal fixation; TAH: total abdominal hysterectomy; AV: arteriovenous

Variables	Total N	RA utilization percentage
TSA	132	6.2%
TKA	1272	4.5%
Carpal tunnel	278	3.2%
ACLR	103	4.6%
Ankle ORIF	221	3.3%
TAH	31	0.6%
AV fistula	97	4.3%

The procedure with the highest rate of RA utilization was total shoulder arthroplasty (5.8%) followed by total knee arthroplasty (4.8%), and the procedure with the lowest rate of RA utilization was total abdominal hysterectomy (1.1%) (p<0.01) (Table 3). The Midwest region has the highest rates of RA utilization for total shoulder arthroplasty (9.9%), total knee arthroplasty (10.9%), anterior cruciate ligament repair (8.1%), and arteriovenous fistula creation (each p<0.01). Notably, there was a disproportionate rate of RA utilization in the Midwest region for total abdominal hysterectomy (16.7%), a higher utilization rate than the Northeast (0.4%), South (2.5%), and West (1.1%) (p<0.01). The West region has the highest rates of RA utilization for carpal tunnel release (11.8%), and a disproportionate rate of RA utilization for ankle open reduction and internal fixation (7.8%) when compared to the Midwest (1.9%), Northeast (1.3%), and South (2.5%) regions (both p<0.01) (Table 4).

**Table 4:** Percent RA utilization by hospital region and surgery. ACLR: anterior cruciate ligament reconstruction; AV: arteriovenous; ORIF: open reduction and internal fixation; RA: regional anesthesia; TAH: total abdominal hysterectomy; TSA: total shoulder arthroplasty; TKA: total knee arthroplasty

Variables	Midwest	Northeast	South	West
TSA	9.9%	0.0%	5.5%	6.2%
TKA	10.9%	0.0%	4.8%	3.1%
Carpal tunnel release	8.1%	0.0%	1.1%	11.8%
ACLR	8.1%	0.0%	2.2%	7.6%
Ankle ORIF	1.9%	1.3%	2.5%	7.8%
TAH	16.7%	0.4%	2.5%	1.1%
AV fistula	6.4%	0.0%	2.1%	6.1%

## Discussion

The main finding in this study was that different hospital regions (i.e., Midwest, Northeast, South, West) had statistically significant differences in RA utilization, both when compared overall by region as well as by procedure. Significant differences in RA utilization rates existed between procedures, such that common orthopedic procedures had the highest rates of RA utilization while non-orthopedic procedures had significantly lower rates of RA utilization. Our study aligned with previous studies in demonstrating that general anesthesia techniques remained the primary anesthetic of choice for most common RA use cases [16], despite increasing literature demonstrating the benefits of RA [19,20].

The South region delivered the overwhelming majority of RA overall, delivering 65.9% of all RA in this study population. This is largely because the majority of HCA hospitals were located within this state-based region, with the West region having the next most hospitals per region. This is in stark contrast to the Northeast region, a region in which HCA only has one hospital. When examining the data on a within-region basis, the Midwest had the greatest RA utilization rate, despite only equating to 0.3% of total anesthetics delivered in this database. This contrasted with the South, which comprised 65.9% of delivered RA but on a within-region basis, had a RA utilization rate of 3.6%. These important trends suggest two important takeaways as follows: (1) regions that have high total RA delivery rates may not necessarily have high RA utilization rates, and (2) total delivery rates by hospital region are influenced by the number of hospitals within a given region, as well as the volume of each contributing hospital, among other factors.

Our analysis of RA utilization rates by procedure overall revealed important trends to suggest differences in RA utilization based on procedural type. The top three procedures in terms of RA utilization rates were total shoulder arthroplasty, total knee arthroplasty, and anterior cruciate ligament reconstruction, each representing a common, high-volume orthopedic procedure with applicable upper extremity or lower extremity blocks. A study by Cozowicz et al. in 2016 documented the use of neuraxial anesthesia for total knee arthroplasty between 2006 and 2013 as 24.7-21.3%, and the use of peripheral nerve blocks for TKA during the same time period as increasing from 10.3% to 20.4% [16]. Our data reports a much lower rate of RA utilization for TKA (4.5%), which may be attributable to various factors including hospital urbanicity [16,21], resource limitations [12], and physician training and comfort level [22].

For both total shoulder and knee arthroplasty, the Midwest region, which is responsible for delivering the third most RA behind the South and West, had the highest utilization rate (2.8% and 6.1%, respectively). The Midwest also had the highest RA utilization rate for anterior cruciate ligament reconstruction, total abdominal hysterectomy, and arteriovenous fistula creation, suggesting it had the highest overall mean RA utilization rate. We believe this represents an important trend in RA utilization that should be tested across various other databases. A similar finding is that the West had a statistically significant increase (mean difference +3.7%) in RA utilization rates for carpal tunnel release (11.8%) compared to other regions. We felt that local infiltration might be a confounder in this relationship, so we excluded it from the analysis, only isolating true block administration. Therefore, this data reflected that the West region had disproportionate RA utilization rates for both carpal tunnel release and ankle open reduction and internal fixation, underlining important practice trends.

Limitations

This study is not without limitations. The retrospective nature of this study is a major limitation. The findings in the studies were limited to associations and do not identify any causal relationships given the limitations in study design. Secondly, the mean RA utilization rates in our study were just meant across the timeline included in the study. However, based on previous studies, we know that RA utilization rates may significantly change, i.e., decrease or increase over time. Therefore, in future studies, it will be necessary to better define these trends by analyzing RA utilization rates on a per-year basis. Third, although univariate statistics allowed us to identify important trends in data, we were unable to perform further analyses and stratify our data by hospital urbanicity and other possible factors. Lastly, these data are comprised of HCA-hospital data across the nation. They are from one hospital system; therefore, they do not represent a remarkably heterogenous group of hospitals (e.g., academic, private, safety net, etc.) and instead represent the HCA private hospital network. We, therefore, do not know the exact type, size, or number of hospitals included in the sample. In addition, there may be other confounding variables not included in our analysis, including patient comorbidity status or severity of surgical diagnosis.

## Conclusions

Significant variations in RA utilization rates were found by region within the United States, with the West having the highest utilization for ankle open reduction and internal fixation and carpal tunnel release, and the Midwest having the highest rate for total knee arthroplasty, anterior cruciate ligament reconstruction, total abdominal hysterectomy, and arteriovenous fistula creation. Future studies should consider using a similar statistical approach in other national databases to see what differences in RA utilization rates exist.
